# Sterolomics: State of the art, developments, limitations and challenges^☆^

**DOI:** 10.1016/j.bbalip.2017.03.001

**Published:** 2017-08

**Authors:** William J. Griffiths, Yuqin Wang

**Affiliations:** Swansea University Medical School, Singleton Park, Swansea SA2 8PP, UK

**Keywords:** Sterol, Cholesterol, 7-Dehydrocholesterol, Oxysterol, Free radical reaction, Mass spectrometry

## Abstract

Sterolomics can be thought of as the quantitative determination of the entire complement of molecules based on the cyclopentanoperhydrophenanthrene skeleton in a system. Mass spectrometry is the dominant analytical technology employed. In this article we highlight some pitfalls in analysis, data interpretation and annotation. We give our opinion on how some of these pitfalls can best be avoided. This article is part of a Special Issue entitled: BBALIP_Lipidomics Opinion Articles edited by Sepp Kohlwein.

## Highlights

• Pitfalls in sterolomic analysis and data interpretation
• Care needed to avoid ex vivo oxidation
• Dangers with databases and purity of authentic standards

## 1 Introduction

Based on the Lipidmaps classification system [1], sterolomics can be thought of as the quantitative determination of the entire complement of molecules built on the cyclopentanoperhydrophenanthrene skeleton in a cell type, tissue, organ, or body fluid. This definition includes oxysterols, hormonal steroids, bile acids and their precursors and metabolites. The complexity of the sterolome is magnified when the existence stereochemical isomers is taken into consideration. For simplicity, in this article we will concentrate on mammalian oxysterols derived primarily from cholesterol. For those interested in steroid hormone, bile acid or vitamins D analysis excellent articles can be found in the textbook “Steroid Analysis” edited by Makin and Gower [2]. A useful collection of articles more specifically on steroid hormones can be found in a 2016 special issue of the Journal Steroid Biochemistry and Molecular Biology [3] and on vitamins D in another 2016 special issue of the same journal [4], [5]. Clayton and Griffiths and Sjövall have written authorities reviews on bile acids and their analysis [6], [7].

Mass spectrometry (MS) is the dominant analytical technology employed in sterolomics. Most sterolomic studies have been performed on human plasma or serum with fewer studies performed on other biofluids e.g. cerebrospinal fluid, or tissues e.g. brain [8]. When data is compared from different studies reported in the literature it is important to establish whether the report refers to “total” sterol levels where an alkaline hydrolysis step has been performed to hydrolyse sterols esterified with fatty acids or whether “free” non-esterified sterols are the subject of investigation. In general studies performed using gas chromatography (GC)–MS include an alkaline hydrolysis step while those which utilise liquid chromatography (LC)-MS may or may not include saponification. The gold standard methods for oxysterol analysis is by GC–MS and includes a saponification step [9].

## 2 Experimentation

For oxysterol analysis nearly all methods require an extraction step followed by a separation step. The most popular extraction methods are based on Folch-like extractions using methanol and chlorinated solvents, however, our preference is for the single-phase extraction of non-esterified oxysterols is into ethanol, which will also extract oxysterols esterified with sulphuric acid or acetal linked sugars [8], [10]. The choice of extraction solvents should be made based on any subsequent requirement for a saponification step and the stability of the target analytes. Oxysterols with a 7-hydroxy-3-oxo-4-ene function are labile and will dehydrate in basic solutions, while oxysterols derived from 7-dehydrocholesterol (7-DHC) with a 5,7-diene structure are also susceptible to reaction in basic solutions. It is important for those new to the oxysterol field to be aware that not only cholesterol, but also its precursors, particularly desmosterol and 7-DHC can be the parent sterol from which oxysterols are derived, both in vivo and ex vivo.

A major concern in all oxysterol analysis is uncontrolled ex vivo reaction of cholesterol, and also desmosterol and 7-DHC, with atmospheric oxygen to generate oxysterols which have similar structures to those formed in vivo [11], [12]. As cholesterol is usually more than 1000 fold more abundant in biological samples than endogenous oxysterols only a minor degree of ex vivo oxidation can cause extreme confusion upon data interpretation. 7-DHC is more than 100 times more reactive towards free radical oxidation than cholesterol and it propensity towards ex vivo oxidation can be a source of further confusion [13]. It is imperative to minimise these unwanted events. This is often achieved by the addition of antioxidants like butylated hydroxytoluene (BHT) and or triphenylphosphine (TPP) [9], [12]. Our preference to avoid confusion over ex vivo oxidation of cholesterol, 7-DHC and other sterols, is to separate more polar oxysterols from more hydrophobic sterols like cholesterol and 7-DHC in the first step of sample handling following extraction [8], [10], [14]. An efficient method to monitor ex vivo oxidation during sample handling is to add deuterated cholesterol and/or 7-DHC during the extraction step and monitor the formation of deuterated oxysterols [12], [15].

To make meaningful biological conclusions based on oxysterol analysis it is imperative to make quantitative or at least semi-quantitative measurements. This is best performed by the addition of stable-isotope labelled internal standards during the extraction step [9]. Ideally a labelled analogue should be included for each target analyte. This may not be possible, in which case an isotope-labelled version of a structurally similar analyte is the next best option. A major source of inter-laboratory variability in quantitative measurements, even when identical methods are employed, can be derived from variability in the purity of unlabelled standard compounds. The response of the isotope labelled standard is usually calibrated against known amounts of “pure” unlabelled compound. But how pure is a “pure” unlabelled compound? Can the analyst trust the purity indicated by the vendor, which is perhaps based on thin layer chromatography? Our preference is to purchase quantitative standards from Avanti Polar Lipids Inc. where exact amounts of standard are dissolved in a defined volume of solvent and a certificate of analysis is provided showing purity data.

Most oxysterol analysis based on GC–MS utilises selected ion monitoring (SIM) where the intensity of one or just a few ions are measured and analyte identification is dependent on GC retention time. A similar approach is used in LC–tandem MS (MS/MS) methods where multiple reaction monitoring (MRM) is employed for detection and LC separation relied upon for identification [16]. These methods are perfectly valid, but do rely on the earlier analysis of appropriate standards to define chromatographic retention times. Problems arise when there is co-elution of compounds which the analyst is unaware of. A disadvantage of both SIM and MRM methods is that unexpected compounds will not be detected unless the appropriate *m*/*z* channel is included. Even if an unexpected compound is detected it cannot be identified by SIM or MRM in the absence of an authentic standard. This problem can be overcome in GC–MS by recording a full scan electron ionisation mass spectrum which will be rich in structural information. In LC-MS analysis, our preference it to exploit derivatisation chemistry which will provide information on functional groups and also to utilise multistage fragmentation (MS^n^) to maximise the information provided by MS [8], [10], [14].

There are a huge number of isomeric oxysterols, for instance in human plasma 4β-, 7α-, 7β-, 22R-, 24S-, 25- and (25R)26-hydroxycholesterols are found. The situation is further complicated in cell cultures where 24R- and (25S)26-isomers can also be present and is much more complicated for dihydroxycholesterols where there are two sites of variability. Thus, for secure identification and quantification, chromatographic separation prior to MS analysis is a pre-requisite. However, if the analyst is certain of the nature of a dominant oxysterol in a particular matrix a shotgun method in the absence of chromatography could be employed e.g. for 24S-hydroxycholesterol in mammalian brain.

When different laboratories analyse the same sample for a pre-defined list of oxysterols the spread of values determined can be quite alarming. For example, in 2014 eleven laboratories measured the “total” 24S-HC content of two serum samples by MS methods, the mean ± standard deviation values for the two samples were 60.9 ± 36.7 ng/mL and 38.0 ± 21.6 ng/mL. The median values with the 16%–84% range given in brackets were 57.0 (38.5–78.9) ng/mL and 33.0 (23.7–48.6) ng/mL [17]. Although, most values clustered about the median, work still needs to be done to harmonise analytical methods. Our belief is that inter-laboratory reproducibility will be best improved by harmonising the use of quantitative standards.

## 3 Databases

While databases are very useful, they can be very dangerous when used without careful consideration. Attempting to simply match a measured mass, however accurately determined, to a compound in a database can lead to gross misinterpretation of results. This is particularly true in the field of oxysterols where multiple isomers exist. By simply tabulating the arbitrary “top-hit isomer” cannot only mislead the reader but also cause problems when published data is extracted in an automated fashion. We recommend the use of the LipidMaps data base http://www.lipidmaps.org/data/structure/index.html which not only provides exact mass values, structures in an easily exported form, systematic names but also InChIKey codes for defining structural certainty. Links can also be found to other databases such as the Human Metabolome Data Base (HMDB), LipidBank and KEGG. With respect to lipid annotation from MS data Liebisch et al. have developed a hierarchical system for lipid annotation based on structural information evident from the analysis [18].

## 4 Limitations and future challenges

What are the current limitations to oxysterol analysis? As mentioned above, a major limitation is the discrepancy in inter-laboratory measurements. We encourage the community, spearheaded by the European Network for Oxysterol Research (ENOR) to further engage in quality assessment schemes. Collaboration with a chemical supplier, such as Avanti Polar Lipids Inc., in a similar manner to that exploited by the LipidMaps consortium, could be of great benefit in this regard.

A major challenge for researchers analysing oxysterols is to resolve different isomers. When a value is reported for 24S-HC in a biological sample, is the researcher sure he/she is measuring 24S-HC or a combination of both 24S- and 24R-isomers? While 24S-HC is undoubtedly the dominant isomer in human plasma, the 24R-isomer can be formed by many different primary cells, and is also found in e.g. mouse plasma [19]. The problem of chromatographically overlapping isomers can be even more extreme when dihydroxycholesterols are considered.

Despite these words of caution analytical methods based on MS are continually being refined and improved. A very exciting area of new research is oxysterol mass spectrometry imaging (MSI). Using derivatisation and MSI it has been possible to localise 7-oxocholesterol in brain [20].

## 5 Concluding remarks

Oxysterols were once thought of as uninteresting intermediates in the metabolism of cholesterol to steroid hormones and bile acids. An increasing volume of data has now shown that these molecules can be ligands to nuclear receptors and G protein-coupled receptors and through binding to the protein INSIG regulate cholesterol biosynthesis via SREBP-2 [21]. As biologists and clinical scientists become more aware of oxysterols there becomes a greater demand for their careful analysis.

## Transparency document

Transparency document. Image 1

## Conflict of interest

The authors declare no conflict of interest.
